# Tinea barbae following ruxolitinib cream application in a vitiligo patient: a case report

**DOI:** 10.3389/fmed.2026.1765822

**Published:** 2026-05-08

**Authors:** Maurizio Romagnuolo, Luisa Carlotta Rossi, Gianluca Tavoletti, Francesca Germiniasi, Anna Grancini, Chiara Moltrasio, Angelo Valerio Marzano, Silvia Alberti-Violetti

**Affiliations:** 1 Dermatology Unit, Fondazione IRCCS Ca' Granda Ospedale Maggiore Policlinico, Milan, Italy; 2 Department of Medical Biotechnologies, University of Siena, Siena, Italy; 3 Microbiology and Virology Unit, Fondazione IRCCS Ca' Granda Ospedale Maggiore Policlinico, Milan, Italy; 4 Department of Pathophysiology and Transplantation, Università degli Studi di Milano, Milan, Italy

**Keywords:** adverse event, infection, ruxolitinib cream, tinea, vitiligo

## Abstract

Ruxolitinib 1.5% cream, a topical Janus Kinase (JAK) 1/2 inhibitor, represents a novel targeted treatment for non-segmental vitiligo, a common acquired depigmentation disorder of autoimmune origin clinically characterized by milky-white patches on the affected body areas. The efficacy of topical ruxolitinib in vitiligo has been demonstrated in clinical trials and real-life observation, with an overall safety profile. Mild-to-moderate reported adverse events include site-application acne, nasopharyngitis, and site-application pruritus, while increased susceptibility to viral skin infections, due to its mechanism of action leading to local immunosuppression, remains debated. No data are currently available on the potential susceptibility to fungal infection. We present here a case of a 53-year-old man who developed tinea barbae by *Trichophyton tonsurans* following ruxolitinib application, which eventually led to permanent treatment discontinuation.

## Introduction

Vitiligo is a chronic autoimmune depigmentation skin disorder characterized by the acquired loss of functional epidermal melanocytes and clinically by milky-white, non-scaling patches in affected areas (1). With an estimated prevalence of 0.5 to 2% of affected individuals worldwide, it represents one of the most common dermatological diseases and, although asymptomatic, is associated with considerable quality of life deterioration mainly due to aesthetic unease, its prolonged and unpredictable course, and the lack of effective and durable repigmentation therapies (2). The recent approval of 1.5% ruxolitinib cream, a topical Janus Kinase (JAK) 1/2 inhibitor, as the first targeted and effective therapy for non-segmental vitiligo (NSV) in adults and adolescents >12 years old represented a milestone for vitiligo treatment, also reflected by patients’ quality of life improvement (3, 4). Topical ruxolitinib is generally safe and well-tolerated, with no major infection risk reported during clinical trials, although real-life data should be further gathered to corroborate the trial’s findings. We present here the case of a patient affected by facial vitiligo who developed tinea barbae during topical ruxolitinib treatment.

## Case description

**Figure 1:**
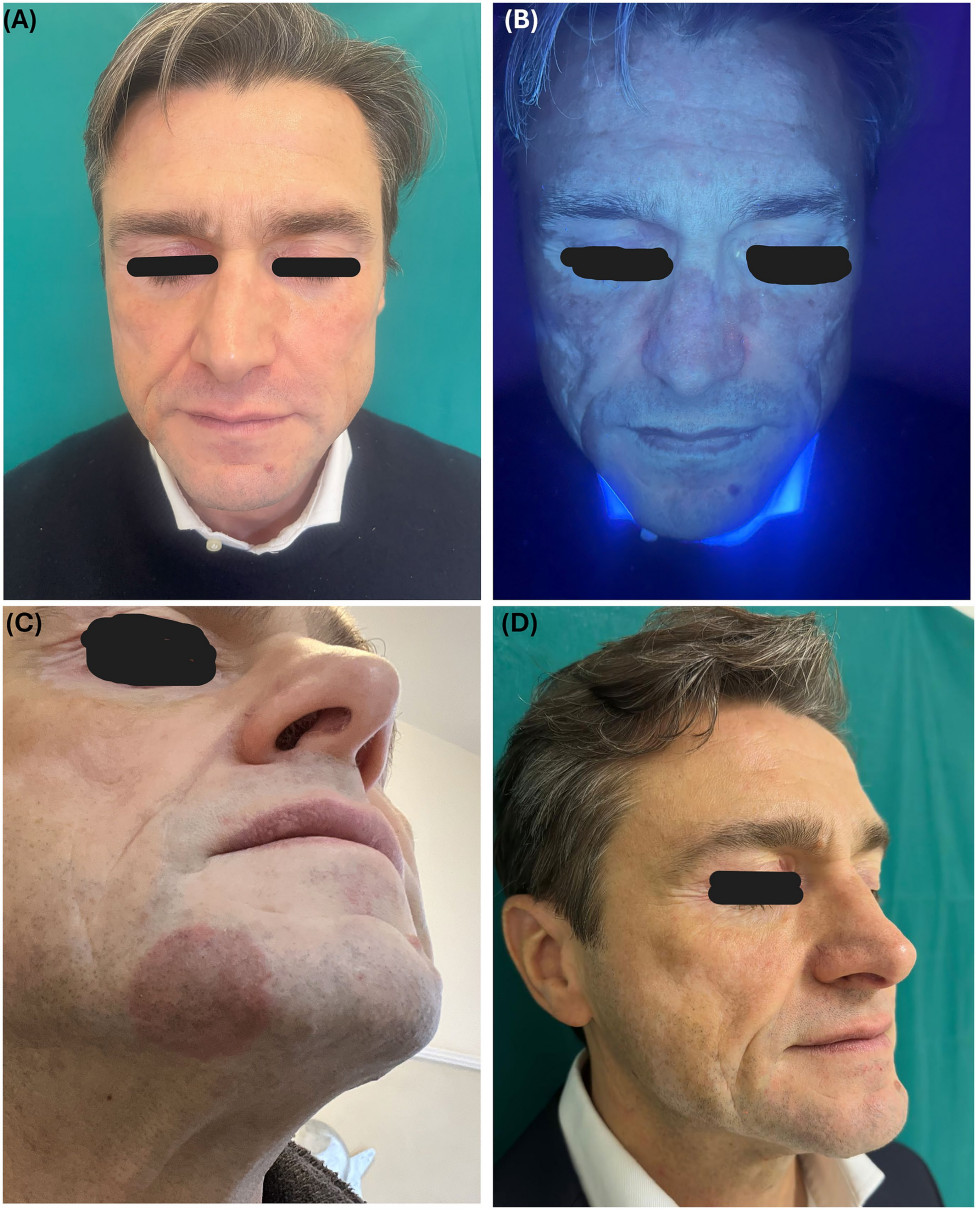
Baseline clinical photographs of vitiligo lesions on the patient’s face under normal light **(A)** and Wood’s lamp examination **(B)** (December 2024); first episode of tinea barbae caused by *T. tonsurans* presenting as round erythematous and slightly scaling patch on the right mandibular area **(C)** (February 2025); clinical remission of the tinea and persistence of vitiligo at the last follow-up **(D)** (October 2025).

A 53-year-old white male came to our attention with a history of longstanding non-segmental vitiligo (disease onset at 14 years old) affecting the face, hands, and elbows. Patient’s past medical history was unremarkable except for an autoimmune hyperthyroidism currently treated with levothyroxine and arterial hypertension managed with olmesartan medoxomil 20 mg per day. A positive family history of autoimmune diseases was reported, namely alopecia areata (mother) and vitiligo (maternal aunt). Upon clinical and Wood’s lamp examination, depigmented patches were clearly visible in the above-mentioned areas (face, hands, and elbows) with a total body surface area (BSA) of 2.82% and a facial BSA of 2.25% calculated with the Vitiligo Extent Score (VES) tool (Figures 1A,B) (5). Based on the vitiligo clinical extent and the reported patient’s discomfort, ruxolitinib cream 1.5% was prescribed for two daily applications on the face (December 2024). However, after 2 months of follow-up, in February 2025, the patient reported the onset of a mild pruritic lesion on the face: Clinical examination revealed a circular erythematous, slightly infiltrated, and desquamative patch on the right cheek with scattered papular lesions centered on hair follicles (Figure 1C); no pustules, nodules, or regional lymphadenopathy were detected. Dermoscopy of the lesion was not performed. In the clinical suspicion of a superficial dermatophyte infection of the beard area, skin scraping was performed, revealing fungal hyphae at extemporary microscopic examination using a 10% potassium hydroxide mount. In addition, microbiological culture using two different agar media, Sabouraud plus gentamicin and chloramphenicol (SGC2 bioMérieux®) and Sabouraud plus chloramphenicol and cycloheximide (Becton Dickinson®) stored at 28 ± 2 °C led to the formation, after 20 days, of white-to-yellowish flat powdery colonies, subsequently identified by matrix-assisted laser desorption/ionization time-of-flight (MALDI-TOF) as *Trichophyton tonsurans*. The patient denied any contact with animals, pets, or affected individuals in the last few months, although frequent job travel for work was reported. We decided to stop topical ruxolitinib application, and an antifungal therapy consisting of itraconazole 100 mg daily and terbinafine cream (1 application daily) was prescribed for 8 weeks, leading to clinical remission of the tinea, and ruxolitinib cream was restarted for the vitiligo (April 2025). The choice of a low-dose systemic treatment combined with a different-class topical antifungal was made due to the reported gastrointestinal intolerance of the patient to antimicrobial drugs. However, in July 2025, a new circular lesion appeared in the same area of the right cheek, which again revealed fungal hyphae upon microscopic examination following skin scraping. A new augmented 8-week cycle of antifungal therapy consisting of itraconazole 100 mg two times per day and terbinafine cream application twice per day was prescribed, and topical ruxolitinib was resuspended. During his last follow-up, in October 2025, clinical remission of the tinea was observed (Figure 1D), and ruxolitinib cream reinitiation was proposed, but the patient preferred to permanently discontinue ruxolitinib application, worrying about a tinea recurrence and the reported side effects of the antifungal therapy, in particular asthenia and nausea. No repigmentation was observed in the treated areas, probably due to the short and inconstant application of ruxolitinib.

## Discussion

Topical ruxolitinib represents the first pathogenesis-driven approved treatment for NSV with a clear efficacy on facial repigmentation: approximately one third of the patients enrolled in the clinical studies TRuE-V1 and TRuE-V2 reached the primary endpoint of facial-Vitiligo Area Scoring Index (f-VASI) 75 at week 24 (75% reduction of vitiligo extent compared to the baseline) (4). Moreover, the same studies showed progressive clinical improvement with long-term ruxolitinib use, with 50.8% of patients reaching f-VASI75 at 52 weeks (6). Regarding safety, treatment-related side effects reported in the trials were application-site acne, nasopharyngitis, and application-site pruritus, all classified as mild-to-moderate, while the only notified infective adverse events were oral herpes, which occurred in approximately 1% of the patients in each arm, with no significant differences between treatment and vehicle groups (4, 7). Viral skin infections, including herpes simplex, herpes zoster, and molluscum contagiosum, were reported in 17 patients (1–1.8%) applying ruxolitinib cream in the atopic dermatitis trial (8). A recent analysis of the FDA adverse events reporting system (FAERS) database showed a slightly increased risk of herpes zoster reactivation with topical ruxolitinib use (9). Only a single case report documented flat wart development on the dorsum of the hand during topical ruxolitinib application, which resolved after ruxolitinib discontinuation and with epigallocatechin gallate and polydatin gel as adjuvant treatment (10).

Infection risk could be explained by ruxolitinib’s mechanism of action: JAK1/2 blockade directly inhibits downstream interferon gamma (IFN-γ) signaling, which is a crucial cytokine for antigen recognition, antigen processing, and antimicrobial activity, thus favoring susceptibility to skin infections (3, 11). While no trial and real-world data are available on the association between ruxolitinib application and fungal infections, it is well-known that systemic or local immunosuppression represents a risk factor for mycoses, and IFN-*γ* plays a crucial role in the eradication of dermatophytes (12). Specifically, both T helper (Th)1 with IFN-γ production and Th17 differentiation with IL-17-mediated immune responses are considered the major key drivers in dermatophyte infection control, leading to phagocyte recruitment and activation against fungi (13, 14). Systemic inhibition of JAK1 with oral ruxolitinib, with subsequent downregulation of macrophage activation and T-helper differentiation, has been linked to atypical dermatophytosis and invasive fungal infections in real-world studies (15, 16).

It is plausible that, in our case, ruxolitinib application could have favored tinea barbae proliferation, given the temporal relationship between the events and the lesion localization, although a simple coincidence cannot be excluded, implying that a direct causal relationship cannot be established. *Trichophyton tonsurans*, the etiologic agent identified in our patient, is an anthropophilic dermatophyte regarded as an emerging pathogen for tinea capitis and tinea barbae, especially in males; epidemiological data of recent outbreaks in European countries indicate that attending barber shops could lead to indirect dermatophyte transmission following skin contact with hairbrushes, combs, razors, and shavers (17–19). This observation is in line with the history of our patient, who denied contact with animals or infected individuals but used different shaving kits for his frequent job travels. It remains unclear if tinea recurrence was due to an insufficient antimycotic therapy leading to the incomplete eradication of the dermatophyte, combined with the possible deep colonization of hair follicles or a re-infection with contaminated shaving tools; based on clinical observations, the role of topical ruxolitinib as an immunosuppressive trigger could only be hypothesized also in tinea recurrence.

In conclusion, we present a case of a patient who developed tinea barbae by *T. tonsurans* at the application site of ruxolitinib cream, which led to a permanent discontinuation of therapy based on the patient’s preferences, although a direct causal relationship could not be established between the events. Further real-world data are needed to establish an association between topical ruxolitinib and dermatophyte infection risk in vitiligo patients. Dermatologists should be aware of any reported side effects during topical ruxolitinib treatment, including skin infections, providing effective preventive or management strategies to strengthen treatment adherence and avoid therapy discontinuation.

## Data Availability

The original contributions presented in the study are included in the article/supplementary material, further inquiries can be directed to the corresponding author.
